# Supplementary material for: Machine learning/molecular dynamic protein structure prediction approach to investigate the protein conformational ensemble
Source: Sci Rep. 2022 Jun 15;12:10018. doi: 10.1038/s41598-022-13714-z (PMC9200820; doi:10.1038/s41598-022-13714-z)
Supplement: Supplementary file 1 — Supplementary Information. [file 41598_2022_13714_MOESM1_ESM.pdf]

Supporting Information

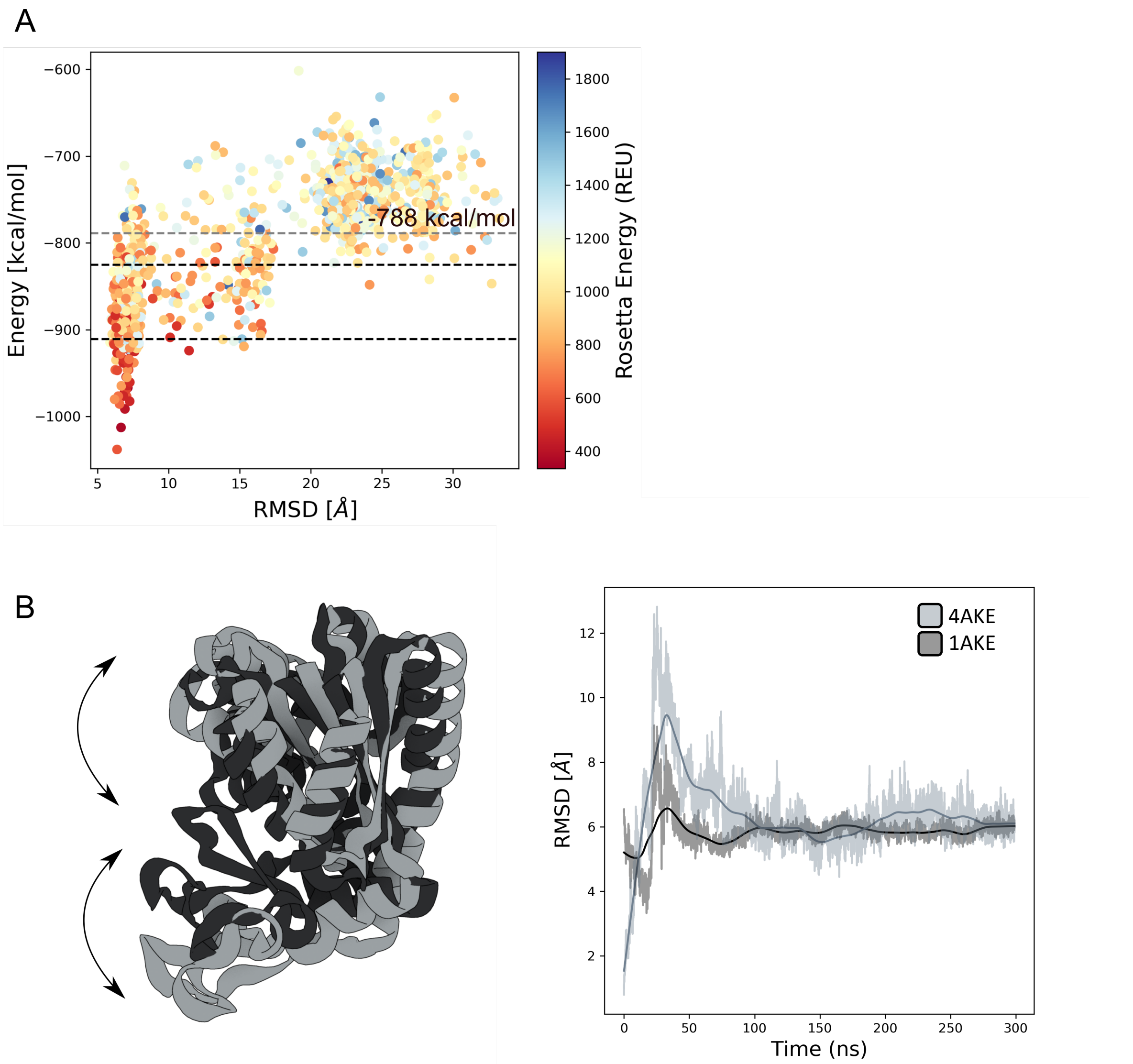

Figure S1. Adenylate kinase A. AWSEM force field vs RMSD profile plot. Each dot represents a protein prediction scored with AWSEM force-field and colored by the Rosetta energy score function ref15. The grey dashed line is the Energy mean filter. The black dashed line represent the X-ray structures energy value; **B.** left panel: overlap between the X-ray structure; right panel: Root-mean-square deviation (RMSD) plot of the 4AKE (light-grey) and 1AKE (dark-grey) protein during molecular dynamics simulation. The solid line represents the regression line relative to the raw data. The images were generated using 3D protein imaging webserver (https://3dproteinimaging.com/protein-imager/).

A

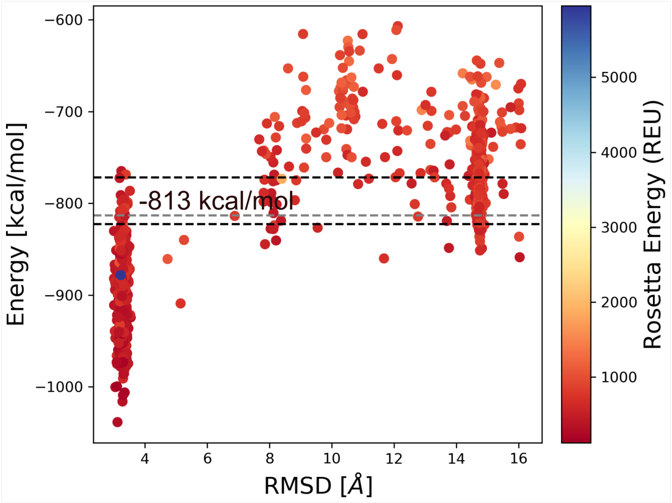

B

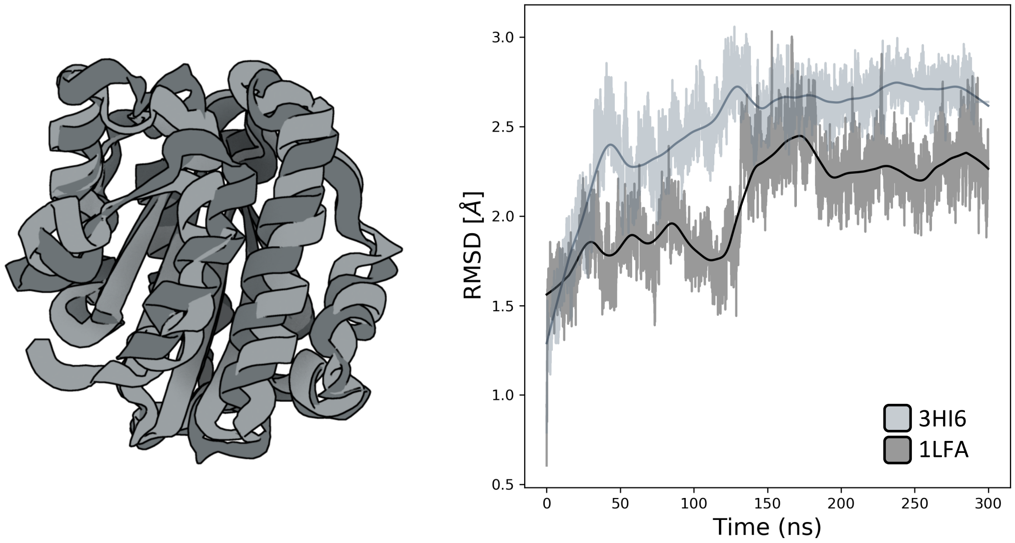

Figure S2. αI-domains of LFA-1. A. AWSEM force field vs RMSD profile plot. Each dot represents a protein prediction scored with AWSEM force-field and colored by the Rosetta energy score function ref15. The grey dashed line is the Energy mean filter. The black dashed line represent the X-ray structures energy value; **B.** left panel: overlap between the X-ray structure; right panel: Root-mean-square deviation (RMSD) plot of the 3HI6 (light-grey) and 1LFA (dark-grey) protein during molecular dynamics simulation. The solid line represents the regression line relative to the raw data.

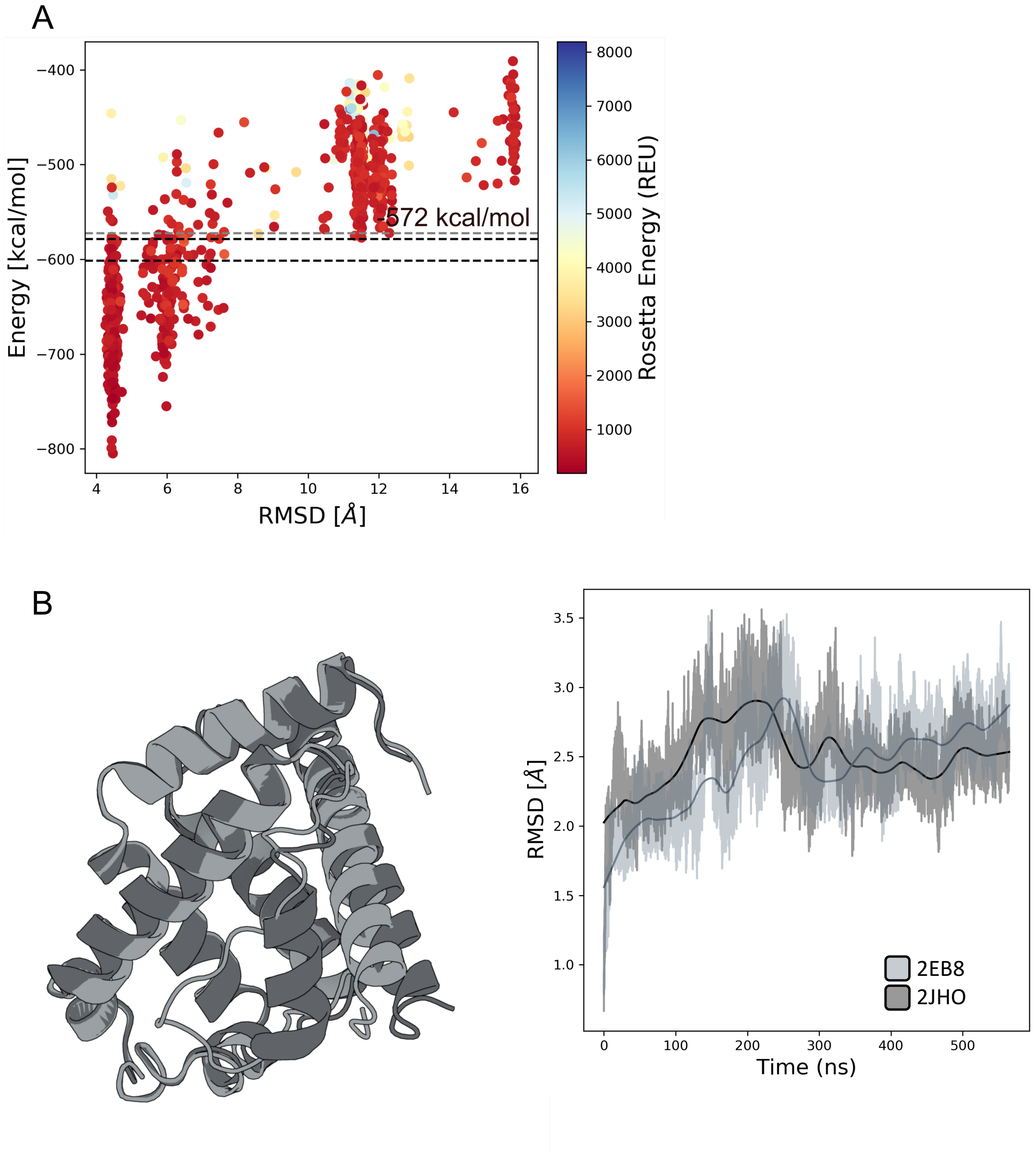

Figure S3. Myoglobin protein. A. AWSEM force field vs RMSD profile plot. Each dot represents a protein prediction scored with AWSEM force-field and colored by the Rosetta energy score function ref15. The grey dashed line is the Energy mean filter. The black dashed line represents the X-ray structures energy value; **B.** left panel: overlap between the X-ray structure; right panel: Root-mean-square deviation (RMSD) plot of the 2EB8 (light-grey) and 2JHO (dark-grey) protein during molecular dynamics simulation. The solid line represents the regression line relative to the raw data. The images were generated using 3D protein imaging webserver (https://3dproteinimaging.com/protein-imager/).

A

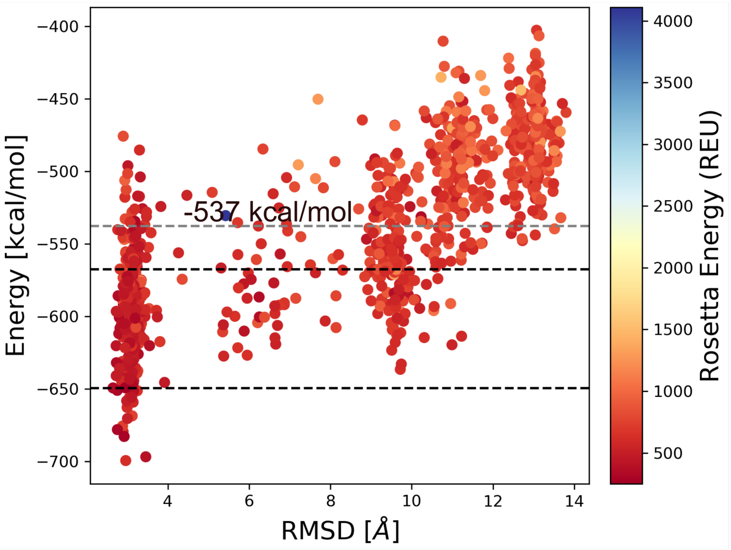

B

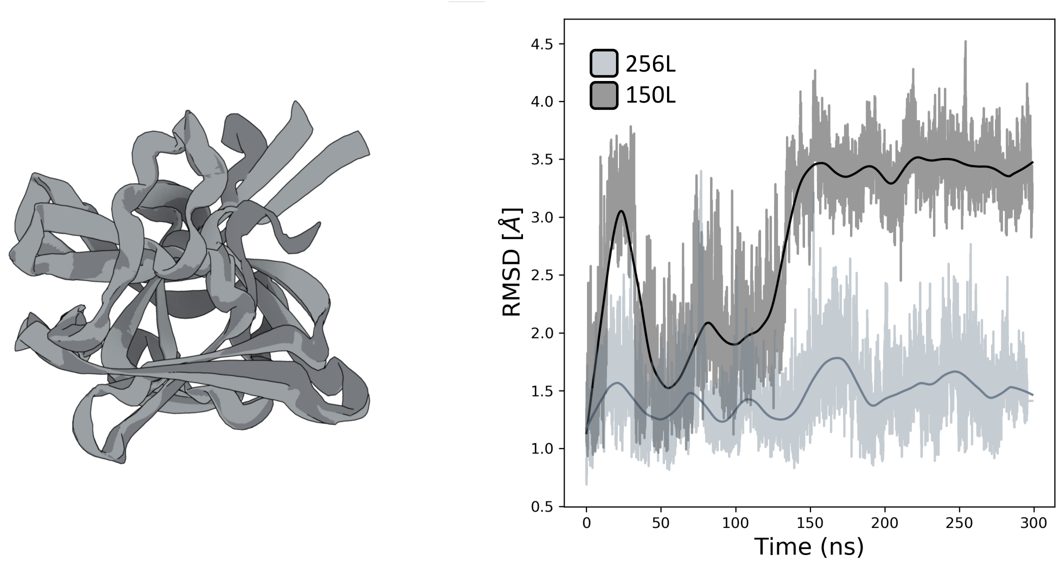

Figure S4. T4 lysozyme. A. AWSEM force field vs RMSD profile plot. Each dot represents a protein prediction scored with AWSEM force-field and colored by the Rosetta energy score function ref15. The grey dashed line is the Energy mean filter. The black dashed line represents the X-ray structures energy value; **B.** left panel: overlap between the X-ray structure; right panel: Root-mean-square deviation (RMSD) plot of the 256L (light-grey) and 150L (dark-grey) protein during molecular dynamics simulation. The solid

line represents the regression line relative to the raw data. The images were generated using 3D protein imaging webserver (https://3dproteinimaging.com/protein-imager/).

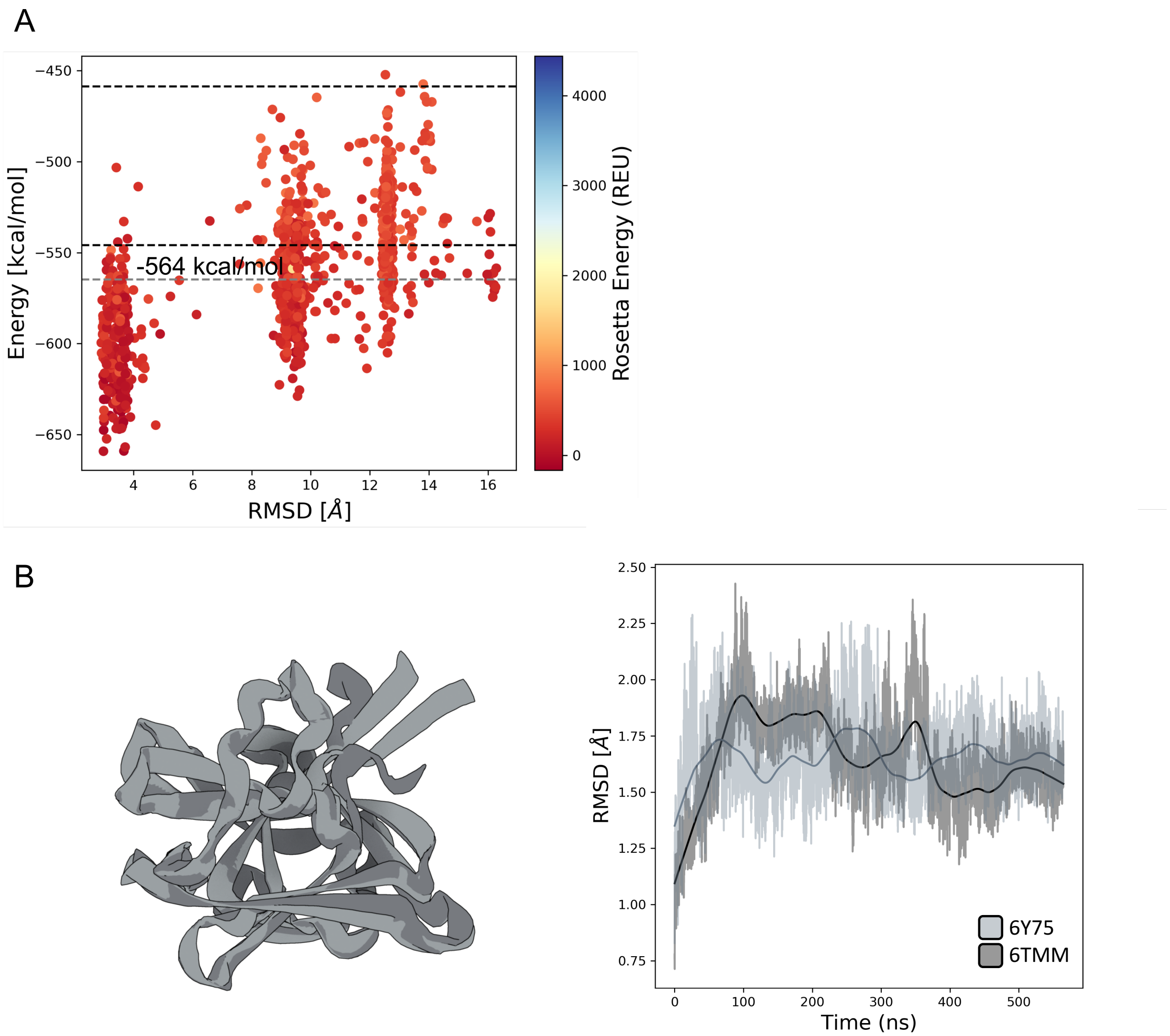

Figure S5. Tetrahymena thermophila-BIL2. A. AWSEM force field vs RMSD profile plot. Each dot represents a protein prediction scored with AWSEM force-field and colored by the Rosetta energy score

function ref15. The grey dashed line is the Energy mean filter. The black dashed line represent the X-ray structures energy value; **B.** left panel: overlap between the X-ray structure; right panel: Root-mean-square deviation (RMSD) plot of the 6Y75 (light-grey) and 6TMM (dark-grey) protein during molecular dynamics simulation. The solid line represents the regression line relative to the raw data. The images were generated using 3D protein imaging webserver (https://3dproteinimaging.com/protein-imager/).

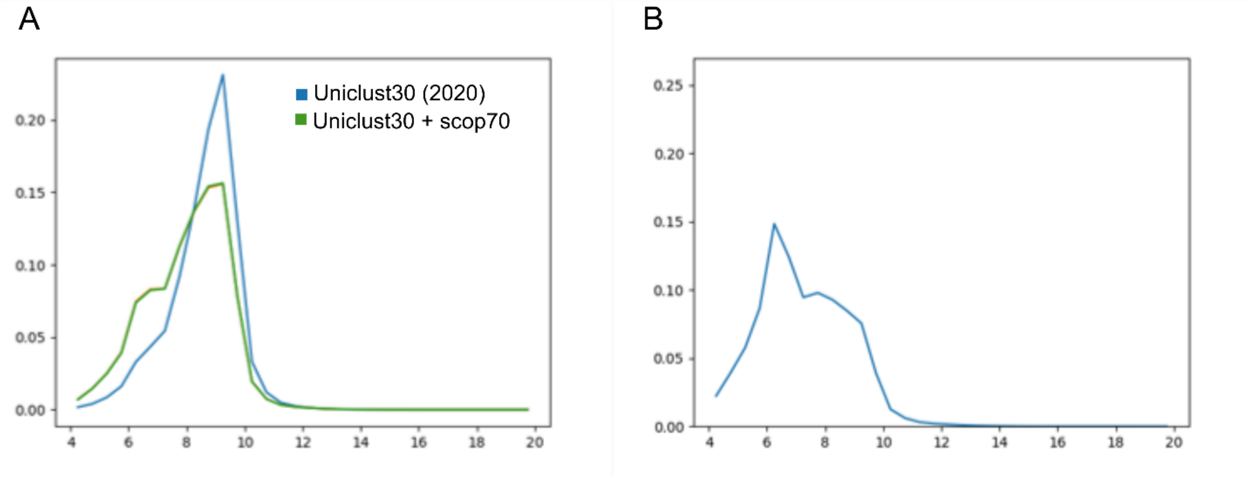

Figure S6. Probability distance distribution: MSA effect. Probability distance distribution between S93 and H98 calculated for the protein structure 2EB8. **A.** Probability distribution plot obtained with trRosetta-MSA protocol using Uniclust30 database (blue) and Uniclust30/scop70 (green). **B**. Probability distance distribution plot obtained with trRosetta-deepMSA protocol using Uniclust30 database (blue).
